# Structural changes in the retina after implantation of subretinal three-dimensional implants in mini pigs

**DOI:** 10.3389/fnins.2022.1010445

**Published:** 2022-09-30

**Authors:** Que Anh Vu, Hee Won Seo, Kwang-Eon Choi, Namju Kim, Yoo Na Kang, Jaemeun Lee, Sun-Hyun Park, Jee Taek Kim, Sohee Kim, Seong-Woo Kim

**Affiliations:** ^1^Department of Ophthalmology, Korea University School of Medicine, Seoul, South Korea; ^2^Department of Ophthalmology, Hanoi Medical University, Hanoi, Vietnam; ^3^Department of Robotics and Mechatronics Engineering, Daegu Gyeongbuk Institute of Science and Technology (DGIST), Daegu, South Korea; ^4^Department of Medical Assistant Robot, Korea Institute of Machinery and Materials (KIMM), Daegu, South Korea; ^5^R&D Center for Advanced Pharmaceuticals and Evaluation, Korea Institute of Toxicology, Daejeon, South Korea; ^6^Department of Ophthalmology, Chung-Ang University College of Medicine, Seoul, South Korea

**Keywords:** retinal prosthesis, subretinal implant, implant design, structural retinal change, three-dimensional microelectrodes

## Abstract

The retinal structural changes after subretinal implantation of three-dimensional (3D) microelectrodes were investigated in a mini pig. Three types of electrode were implanted into the subretinal spaces of nine mini pigs: 75-μm-high 3D electrodes on a 200-μm-thick right-angled polydimethylsiloxane (PDMS) substrate (group 1); a 140-μm-thick sloped PDMS substrate without electrodes (group 2); and a 140-μm-thick sloped PDMS substrate with 20-μm-high 3D electrodes (group 3). One mini pig was used as a control. Spectral domain–optical coherence tomography (SD–OCT) images were obtained at baseline and 2, 6, and 12 weeks post-surgery. Retinal specimens were immunostained using a tissue-clearing method 3 months post-implantation. The 75-μm-high 3D electrodes progressively penetrated the inner nuclear layer (INL) and touched the inner plexiform layer (IPL) 2 weeks post-surgery. At 6 weeks post-operatively, the electrodes were in contact with the nerve-fiber layer, accompanied by a severe fibrous reaction. In the other groups, the implants remained in place without subretinal migration. Immunostaining showed that retinal ganglion and bipolar cells were preserved without fibrosis over the retinal implants in groups 2 and 3 during the 12-week implantation period. In summary, SD–OCT and immunohistology results showed differences in the extent of reactions, such as fibrosis over the implants and penetration of the electrodes into the inner retinal layer depending on different types of electrodes. A sloped substrate performed better than a right-angled substrate in terms of retinal preservation over the implanted electrodes. The 20-μm-high electrodes showed better structural compatibility than the 75-μm-high 3D electrodes. There was no significant difference between the results of sloped implants without electrodes and 20-μm-high 3D electrodes, indicating that the latter had no adverse effects on retinal tissue.

## Introduction

In recent decades, various visual prostheses have been developed to restore vision in patients who have lost their sight due to outer retinal layer degeneration, such as in cases of retinitis pigmentosa or dry age-related macular degeneration (Loudin et al., 2007; Zrenner et al., 2011, 1999; Humayun et al., 2012; Mathieson et al., 2012; Wang et al., 2012; Stronks and Dagnelie, 2014; Ghezzi, 2015; da Cruz et al., 2016; Goetz and Palanker, 2016; Stingl et al., 2017). Clinical outcomes have also shown that use of retinal prostheses may enable vision restoration; however, the level of restoration achieved is relatively low (Humayun et al., 1996; Zrenner et al., 1999; Rizzo et al., 2001, 2003; Caspi, 2009). To overcome this limitation, visual acuity, including spatial resolution, must be improved, and the most common approach involves increasing electrode pixel density. However, the threshold charge density, which depends on the electrode surface area, must be considered when designing high-density electrodes to ensure that it remains below the level that can cause tissue damage (Cohen et al., 2011; Corna et al., 2018). Therefore, geometrically modulating electrode shape to increase electrode area could be an alternative way to accommodate a large number of electrodes within a device of limited size while also minimizing the threshold charge density (Flores et al., 2018).

In order to transduce visual information into electrical signals and conduct them into the retinal tissue, commercially available retinal implants primarily comprise a light-sensing device and a microelectrode array. Using these components, the remaining circuitry of the visual pathway is activated due to electrical stimulation of bipolar and/or retinal ganglion cells (RGCs), depending on whether the corresponding array of electrodes is located suprachoroidally, subretinally, or epiretinally (Margalit et al., 2002). Among these approaches, subretinal prostheses have some advantages such as the strong fixation of the stimulating electrodes and the preservation of the innermost cells of the retina (RGCs), which receive information from the target cells (bipolar cells). Subretinal implantation also can preserve the relatively close distance between the stimulating electrodes and the targeted bipolar cells. Therefore, the stimulating electrodes used to replace lost photoreceptors can stimulate the target cells with the highest efficiency (Mathieson et al., 2012; Wang et al., 2012; Stingl et al., 2013a,^2015^, ^2017^; Lorach et al., 2015a,b; Daschner et al., 2018; Flores et al., 2018, 2019; Ho et al., 2019; Palanker et al., 2020), and various retinal electrodes have been developed with tiny pixels. Daschner et al. (2018) developed a subretinal electrode array with 1,600 pixels (Alpha AMS, Retina Implant AG, Reutlingen, Germany), which is the highest reported pixel density; this array showed satisfactory longevity in clinical trials (Stingl et al., 2017). Photodiode-based two-dimensional (2D) photovoltaic implants with high pixel-densities have also been developed and evaluated both experimentally and clinically (Mathieson et al., 2012; Wang et al., 2012; Ho et al., 2019). Moreover, according to recent reports by Flores et al. (2018) and Ho et al. (2019), a three-dimensional (3D) subretinal implant with a larger electrode surface area could efficiently stimulate non-spiking neurons in the inner nuclear layer (INL). Compared to 2D implants, 3D geometries have advantages such as reduced distance between electrodes and retinal cells and sufficient proximity to target neurons (Mathieson et al., 2012; Flores et al., 2018).

In our previous study (Seo et al., 2019), we reported the fabrication and *in vitro* evaluation of a 3D subretinal electrode array, consisting of a flexible and transparent polydimethylsiloxane (PDMS) substrate and 3-dimensionally protruded electrodes with a height of around 75 μm. The electrodes were deposited with platinum (Pt) to deliver stimulation. Also, the substrate thickness and electrode pitch were fabricated to be 200 and 555 μm, respectively. Our results demonstrated the feasibility of using fabricated 3D microelectrodes as subretinal prostheses. In the next stage of development, it is important to assess the integration of these electrodes with retinal tissue under *in vivo* conditions. Typically, not only the material of a retinal implant but also the design of the substrate and electrodes should be considered when investigating how it will interact with retinal tissue. Thus, the present study investigates post-implantation structural retinal changes for three different subretinal implants with varied geometries over 12 weeks in a mini pig model.

## Materials and methods

### Subretinal implants

To assess the structural retinal changes caused by subretinal implants, three types of implant were designed. The geometries and materials used are summarized in Table 1.

**TABLE 1:** Specifications of the subretinal implants.

Group	Electrode shape	Electrode height (μ m)	Electrode top dimensions or diameter (μ m)	Distance between electrodes (μ m)	Number of electrodes	Substrate material	Total implant height without electrode (μ m)	Total implant size (mm)	Disruption of INL
1	3D	75	70 × 70	555	16	PDMS	246	4.3 × 2.4	Penetrated through the INL
2	N/A	N/A	N/A	N/A	N/A	PDMS	186	5 × 4.5	Within INL
3	3D	20	150	350	98	PDMS	186	5 × 4.5	Within INL

N/A, not applicable.

#### Group 1 (200-μm-thick right-angled polydimethylsiloxane substrate with 75-μm-high three-dimensional electrodes)

**FIGURE 1:**
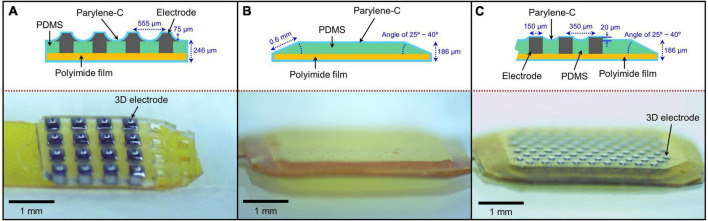
Various subretinal implants used in the assessment of structural retinal changes. **(A)** 75-μm-high three-dimensional (3D) electrodes on a right-angled polydimethylsiloxane (PDMS) substrate, **(B)** a sloped PDMS substrate without electrodes, and **(C)** 20-μm-high 3D electrodes on a sloped PDMS substrate.

The group 1 subretinal implant consisted of 3D silicon electrodes with a PDMS substrate (Figure 1A; Seo et al., 2019). The 3D electrodes protruded approximately 75 μm from the PDMS substrate. The top and bottom dimensions of the electrodes were 70 μm × 70 μm and 200 μm × 200 μm, respectively. The distance between the centers of two neighboring electrodes was 555 μm. The cross-section of the PDMS substrate was rectangular in shape with an angle of 90° and a thickness of 200 μm. The substrate was attached to a 40-μm-thick polyimide film and the entire structure was coated with a 3-μm-thick layer of parylene-C to ensure biocompatibility. Therefore, the total height of the implant was approximately 321 (3 + 75 + 200 + 40 + 3) μm at the center of the electrodes and 246 (3 + 200 + 40 + 3) μm in the areas without electrodes.

#### Group 2 (140-μm-thick sloped polydimethylsiloxane substrate without electrodes)

Group 2 implants had no 3D electrodes and consisted of a 140-μm-thick PDMS substrate with sloped sides attached to a 40-μm-thick polyimide film. And then, group 2 was coated with a 3-μm-thick layer of parylene-C. Therefore, the total height of the implant was approximately 186 (3 + 140 + 40 + 3) μm. The slope length and angle at the edge of the implant were approximately 0.6 mm and 25–40°, respectively. The cross-sectional shape of the implant was trapezoidal (Figure 1B).

#### Group 3 (140-μm-thick sloped polydimethylsiloxane substrate with 20-μm-high three-dimensional electrodes)

Similar to group 2, group 3 implants consisted of a 140-μm-thick PDMS substrate with sloped sides but with 3D electrodes, attached to a 40-μm-thick polyimide film. And then, group 3 was coated with a 3 μm thick layer of parylene-C (Seo et al., 2020). Therefore, its total height was approximately 206 (3 + 20 + 140 + 40 + 3) μm at the center of the electrodes and 186 (3 + 140 + 40 + 3) μm in the areas without electrodes. Similar to group 2, the cross-section of the implant was a trapezoid with a slope length and angle of approximately 0.6 mm and 25–40°, respectively (Figure 1C). The 3D electrodes protruded approximately 20 μm from the PDMS substrate.

### Animals and surgical procedure

The implants were inserted into the subretinal space of one eye of nine mini pigs (MICROPIG, APURES Co., Ltd., Pyeongtaek-si, South Korea) for 12 weeks; one mini pig was used as a control for immunohistochemical examinations. For each group, three pigs were used as identified in Table 2. The mean age of the animals was 11.5 ± 2.6 months (range: 9–15 months), the mean weight was 27.5 ± 1.7 kg (range: 25–30 kg), and the mean axial length of the eyes was 19.8 ± 0.8 mm (range: 18.5–21.9 mm).

**TABLE 2:** Mean (± standard error) values of the total retinal layer (TRL) thicknesses over electrode, over substrate, and over substrate edge at 2, 6, and 12 weeks post-implantation on spectral domain–optical coherence tomography (SD–OCT) images.

Group		TRL before surgery (μ m)	TRL over electrode (μ m)			TRL over substrate (μ m)			TRL over substrate edge (μ m)			Comment
					
			2 weeks	6 weeks	12 weeks	2 weeks	6 weeks	12 weeks	2 weeks	6 weeks	12 weeks	
1	Animal #1	191.6 (± 16.4)	N/A	N/A	N/A	N/A	N/A	N/A	N/A	N/A	N/A	Severe fibrosis (Supplementary Figure 3A)
	Animal #2	189.6 (± 12.9)	121.6 (± 42.2)	N/A	N/A	174.8 (± 30.2)	N/A	N/A	104.8 (± 20)	N/A	N/A	Retinal detachment at 12 weeks (Supplementary Figure 3B)
	Animal #3	206.1 (± 13.1)	95.3 (± 21.6)	55.5 (± 13.5)	48.5 (± 37.3)	172.6 (± 44.6)	154.1	134.1	68.8 (± 19.2)	52.2 (± 3.3)	46.5 (± 40.2)	Figures 2A–C and Supplementary Figure 3C
2	Animal #4	232.7 (± 39.1)	151.4 (± 22.3)	93.8 (± 21.9)	88.3 (± 26.1)	151.4 (± 22.3)	93.8 (± 21.9)	88.3 (± 26.1)	191.5 (± 59.9)	152.3 (± 9.8)	117.8 (± 45.3)	Figure 3A
	Animal #5	228.8 (± 13.1)	112 (± 21)	127.8 (± 10.6)	N/A	112 (± 21)	127.8 (± 10.6)	N/A	137 (± 52.6)	129.3 (± 11.1)	N/A	Reversed substrate (Supplementary Figure 5)
	Animal #6	224.3 (± 13.7)	N/A	N/A	N/A	N/A	N/A	N/A	N/A	N/A	N/A	Retinal detachment
3	Animal #7	217.1 (± 19.2)	158.4 (± 19.3)	N/A	N/A	173.1 (± 16.1)	N/A	N/A	158.8 (± 14.9)	N/A	N/A	
	Animal #8	213.1 (± 17.5)	125.8 (± 15.3)	120.5 (± 14.6)	124.8 (± 27)	151.8 (± 23.8)	138.3 (± 29.6)	130.6 (± 29.1)	199.5 (± 32.3)	150.3 (± 37.7)	165 (± 26.1)	Figure 3B
	Animal #9	221.2 (± 18.6)	170 (± 11.1)	N/A	195 (± 18.7)	174.5 (± 23.6)	N/A	100.6 (± 19.1)	217.8 (± 21.2)	N/A	108 (± 42.9)	Implant could not be found at 6 weeks

N/A, not applicable.

Surgeries were performed by a single surgeon (S-WK from Korea University). Under anesthesia, the skins of the animals were disinfected with 5% iodine solution. Then, the head was covered with a surgical drape and positioned with the nose upright. After a lateral canthotomy, a three-port 23-gauge vitrectomy (Associate; Dutch Ophthalmic Research Center B.V., Zuidland, The Netherlands) was performed using an indirect BIOM lens (Oculus BIOM^®^ ready; Oculus Surgical, Inc., Port St. Lucie, FL, USA). Three ports were prepared by inserting valved trocar cannulas into the sclera 3 mm from the limbus on the ventromedial, ventrolateral, and dorsomedial sides. The vitreous was removed using a vitreous cutter while continually supplying a balanced salt solution (BSS; Alcon, Fort Worth, TX, USA). An anterior capsule-saving lensectomy was also performed. After the core vitrectomy, posterior vitreous detachment from the disc was gently induced using a vitreous cutter to avoid an iatrogenic retinal break. Thereafter, a small hole was created by lightly pressing the superior peripheral retina with a 23-gauge viscoelastic cannula, and less than 0.1 cc of a viscoelastic material was injected into the retinal hole to induce focal retinal detachment. Next, a cannula with BSS was inserted into the subretinal cavity, and the BSS was gently injected to increase the size of the retinal detachment. Once the retinal detachment was large enough to include the peripheral retina, a 2.75-mm slit knife was used to make a scleral incision 1.5 mm from the limbus of the dorsolateral or dorsal side. Thereafter, the incision was lengthened to 5 mm.

Diathermy was used at the hole on the detached retina to create a linear tear into which the implants could be inserted. The implants were inserted into the subretinal space using micro-forceps; partial fluid–air–fluid exchange was performed during implant insertion to decrease the height of retinal detachment and prevent the retinal implant from turning over. Usually, the retinal implant could be driven into the visual streak with the force of inertia created by shaking the eye back and forth. To mitigate the risk of iatrogenic retinal tearing, a 23-gauge curved directional laser probe tip (Endo Ocular Laser Probe; Synergetics, Inc., O’Fallon, MO, USA) with an expandable fiber or a moving shaft was used to push the implanted prosthesis forward and adjust its position. After confirming that the subretinal implant was in the desired position, an air–fluid exchange was performed. Endolaser photocoagulation was then performed around the retinotomy site, and the oil tamponade was completed. All port sites and scleral incisions were sutured using 10-0 nylon (Johnson & Johnson, New Brunswick, NJ, USA) to prevent post-operative oil leakage. The detailed surgical techniques for retinal implant placement have been described in a previous study (Choi et al., 2020).

An adverse complication may have a negative effect on the wellbeing of animals. Following the surgery, some possible adverse events may happen such as the death of pigs, a greater level of pain, or retinal detachment during or after subretinal implantation in the operated eye. Therefore, all pigs in our experiments were closely followed up for at least 24 h after surgery. In addition, surgery was done on one eye for each pig to prevent vision impairment. With the normal fellow eye, pigs could have normal life activities.

### Fundus infrared reflectance and spectral domain–optical coherence tomography

A-scan biometry (SW-1000; Suoer, China) was used to measure the axial length of the eyeballs at baseline. Both 55° field-of-view infrared and Spectral domain–optical coherence tomography (SD–OCT) images of the fundus were obtained using the Spectralis OCT system (Heidelberg Engineering GmbH, Heidelberg, Germany). Vertical and horizontal line scans, as well as raster scans (37 B-scans over an area of 16.5 mm × 16.5 mm in a 55° image), were performed at high resolution (1,536 A-scans per B-scan, lateral resolution = 10 μm/pixel in a 55° image). Up to 100 images were averaged in automatic real-time mode to obtain a high-quality mean image. The total retinal layer (TRL) thickness before surgery was measured along a horizontal line perpendicular to the retinal layers in cross-sectional images (Supplementary Figure 1). The over-electrode and over-substrate TRL thicknesses were measured individually at eight points (four points in the central area and one point each in four marginal areas of the implant) at 2, 6, and 12 weeks post-implantation. We also measured the TRL thickness at the substrate edges (group 1) or slopes (groups 2 and 3) in four different areas (at one point on each edge of the implant). The over-electrode TRL thickness was defined as the distance between the center of each electrode and the inner margin of the internal limiting membrane. The over-substrate TRL thickness was defined as the distance between the surface of the substrate (at the center point between two adjacent electrodes) and the inner margin of the internal limiting membrane (Supplementary Figure 2).

### Immunohistochemical examination (tissue clearing method)

As it was not possible to separate the tissue from an implant without damaging the retina, immunohistochemical staining of whole-mount retinas was performed using a tissue-clearing method. Twelve weeks after surgery, the mini pigs were sacrificed, and all eyes were enucleated. After the anterior segment was removed at the vitreous base level, the remaining eye cup (post-segment) was rinsed in phosphate-buffered saline (PBS) to remove as many of the silicone oil bubbles as possible. The eye cup was cut into a 12 mm × 12 mm square centered around the implant and fixed in 4% paraformaldehyde for 15 min. After being washed with 1 × PBS, whole-mount retinas with sclera were permeabilized in 0.5% Triton X-100 in 1 × PBS for 1 day at 37°C. Thereafter, the retinas were incubated with primary antibodies diluted in whole-mount antibody-dilution buffer (0.5% Triton X-100, 4% serum in PBS) for 3 days at 37°C. The retinas were then washed for 3 × 10 min in 1 × PBS and incubated with secondary antibodies in antibody-dilution buffer for 1 day at 37°C. Next, the retinas were washed for 3 × 10 min in PBS and mounted in tissue-clearing mounting medium (Binaree, Daegu, South Korea) with the GCL uppermost on a chamber slide. The primary antibodies and corresponding concentrations used were as follows: Alexa Fluor^®^ 488-conjugated rabbit anti-MAP2 (1:100; Abcam, Cambridge, UK); Alexa Fluor^®^ 647-conjugated rabbit anti-PKC-α (Abcam); and rabbit anti-GFAP (Dako, Santa Clara, CA, USA). The secondary antibody used for immunofluorescence-based detection was goat anti-rabbit f(ab’)2 488 (1:100; Vector Laboratories, West Grove, PA, USA). Nuclei were counterstained using DAPI (Sigma-Aldrich, St. Louis, MO, USA). 3D images of the retinas were obtained using a confocal microscope (A1 upright confocal microscope; Nikon Co., Tokyo, Japan), and the Imaris software was used for data analysis. Using tissue-clearing methods, it was possible to image the entire 3D structure while simultaneously preserving tissue integrity.

## Results

### Spectral domain–optical coherence tomography imaging and total retinal layer thicknesses

**FIGURE 2:**
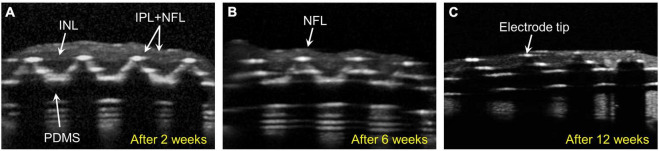
Spectral domain–optical coherence tomography (SD-OCT) images showing retinal changes after the implantation of 75-μm-high three-dimensional (3D) electrodes (group 1). **(A)** The edges of the electrode tips progressively penetrated the inner nuclear layer (INL) and had reached the inner plexiform layer (IPL) 2 weeks after surgery. **(B)** The penetrating 3D electrodes were in contact with the nerve fiber layer (NFL) at 6 weeks. **(C)** At 12 weeks, the tips of the electrodes nearly penetrated the atrophic NFL. The overlying retina remained flat instead of wavy along the contours of the 3D electrodes.

In group 1 (*n* = 3), 75-μm-high 3D electrodes on a substrate with right-angled (90°) edges were implanted into the subretinal spaces for 12 weeks, and post-implantation integration of the implants with the retinal tissue was monitored using SD–OCT. Figure 2 shows the relatively stable integration of the implants with the retinal tissue at 2 weeks post-implantation in all three mini pigs. However, in one mini pig, a severe fibrous reaction was noted around the implant at 6 weeks post-implantation. In another mini pig, retinal detachment was observed at 12 weeks, even though not at 6 weeks. Thus, the implant remained stable under the retina without apparent gross damage in only one mini pig (Supplementary Figure 3). However, SD–OCT imaging also revealed that the 3D electrodes progressively penetrated the INL and touched the inner plexiform layer (IPL). At 6 weeks, the 3D electrodes were in contact with the nerve fiber layer (NFL). At 12 weeks, the tips of the 3D electrodes fully penetrated the NFL, with the overlying retina remaining flat instead of wavy along the contour of the 3D electrodes (Figures 2A–C). Retinal thinning was observed across the implant base but was more obvious at the substrate edge from 2 weeks onward (Supplementary Figure 4). Moreover, TRL thickness measurements obtained using SD–OCT showed trends of constant progressive decrease over the 12-week post-surgery period in group 1. The mean (± standard deviation) TRL thickness over the electrode was 195.7 ± 14.1 μm before implantation (baseline), 108.4 ± 31.9 μm at 2 weeks, 55.5 ± 13.5 μm at 6 weeks, and 48.5 ± 37.3 μm at 12 weeks. The mean TRL thicknesses over the electrode, over the substrate, and over the substrate edge or slope in all nine pigs are summarized in Table 2.

**FIGURE 3:**
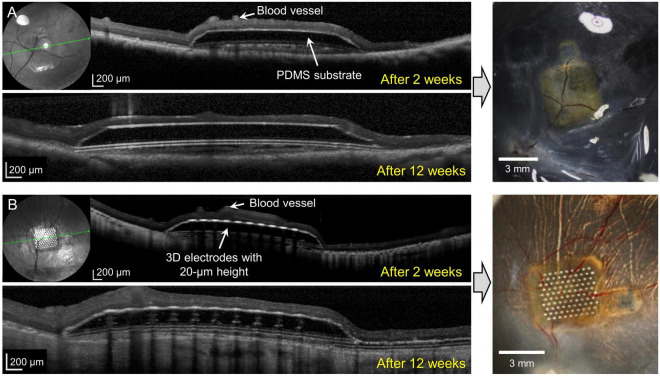
Spectral domain–optical coherence tomography (SD–OCT) images at 2 and 12 weeks after surgery and photographic images after coronal dissection of enucleated eyeballs from groups 2 and 3. **(A)** The implanted polydimethylsiloxane (PDMS) substrate without three-dimensional (3D) electrodes (group 2) remained stable for 12 weeks. Outer retinal layer degeneration was homogeneous over the substrate. The outer nuclear layer (ONL) and photoreceptor layer (PRL) disappeared from 2 weeks onward. The inner retina was relatively intact. **(B)** The 20-μm-high 3D electrodes (group 3) penetrated the lower portion of the inner nuclear layer (INL) and did not reach the IPL. The 3D electrodes were stably integrated into the subretinal space by 12 weeks. The ganglion cell layer (GCL), IPL, and INL remained intact over the electrodes and at the edges of the electrodes throughout the follow-up period of 12 weeks.

In group 2 (implants without any 3D electrodes on a sloped substrate; *n* = 3) and group 3 (implants with 20-μm-high 3D electrodes on a sloped substrate; *n* = 3), on SD–OCT images, the top surfaces of the electrodes were observed to remain within the INL in all mini pigs. In group 2, the implants remained stable in the subretinal space between the retina and RPE over the entire 12-week period. Outer retinal layer degeneration was homogeneous across the implants. On SD–OCT images, the outer nuclear layer (ONL) and photoreceptor layer (PRL) were observed to have been lost from the second week onward; however, the inner retina remained relatively intact. The morphology of the INL and ganglion cell layer (GCL) with NFL did not differ from that of areas away from the implant (Figure 3A). Retinal detachment on one side of the implant was observed in one mini pig in this group at 2 weeks after surgery; however, this phenomenon resolved spontaneously within 1 month. In another mini pig, the PDMS substrate was implanted upside down because of the surgeon’s error; after 2 weeks, the lateral edge of the inverted PDMS substrate on the left side was found to be fitted to the curvature of the retina, and the overlying retinal tissue (GCL and inner retina) was undamaged. The lateral edge on the other side, however, nearly penetrated the retinal tissue (Supplementary Figure 5). Nevertheless, retinal tissue remained stably integrated with the implant at 6 weeks. The TRL thickness over the substrate was 228.6 ± 21.9 μm before surgery (baseline), and 88.3 ± 26.1 μm over the substrate and 117.8 ± 45.3 μm over the substrate slope at 12 weeks.

In group 3, implants were found to be stably integrated in the subretinal space at 2, 6, and 12 weeks after surgery. SD–OCT images showed that the retinal changes were similar to those observed in the outer retinas in group 2. The 20-μm-high electrodes penetrated the lower portion of the INL, but the GCL, IPL, and INL remained intact over the electrode and at the implant edges throughout the 12-week period. The 3D electrodes observed in the INL did not reach the IPL (Figure 3B). OCT images showed that the groups with no electrodes or lower height (20 μm) of electrodes (groups 2 and 3) had less tissue disruption and better integration of the implant with the retinal tissue, which remained stable, when compared with the higher (75 μm) electrodes (group 1). The TRL thickness over group 3 implants was 217.1 ± 18.4 μm before implantation (baseline), and 159.9 ± 22.8 μm over the electrode, 115.6 ± 24.1 μm over the substrate, and 136.5 ± 34.5 μm over the substrate slope at 12 weeks.

**FIGURE 4:**
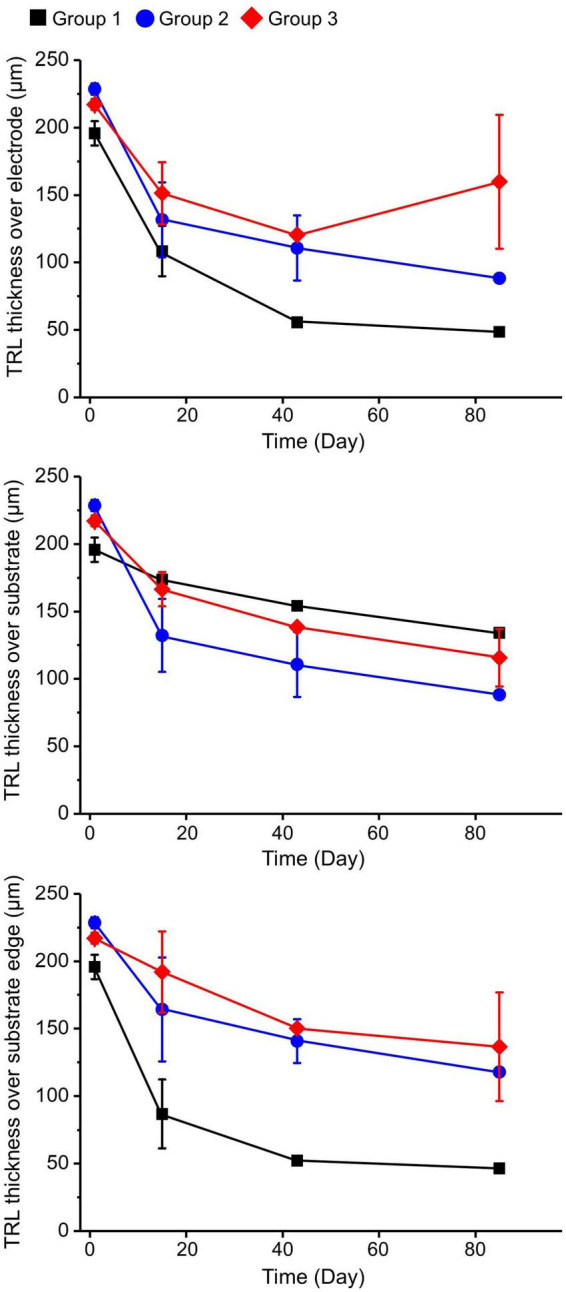
Total retinal layer (TRL) thicknesses over the electrode, over the substrate, and over the substrate edge or slope in each group at 2, 6, and 12 weeks after implantation. On spectral domain–optical coherence tomography (SD–OCT) images, the TRL thicknesses in group 1 showed a trend of constant progressive decrease over time at all measurement points. Although both groups 2 and 3 demonstrated similar trends of decreasing TRL thickness at all measurement points during the 12-week observation period, the degrees of decrease seemed to be less severe than in group 1.

During the observation period, group 1 showed more-severe thinning of the TRL over the electrode and over the substrate edge in comparison with the other groups. At 12 weeks after operation, the TRL thickness over the substrate edge in group 1 was reduced to about 25%, while in groups 2 and 3 it was reduced to about 50% compared to the baseline, implying that the 20-μm-high electrodes with the sloped substrate had less detrimental effects on the retinal tissue than the 75-μm-high electrodes with the 90°-angled substrate edges. However, there was no distinctive difference in the TRL-thickness changes over the substrates of all groups (Figure 4). In addition, there was a relatively small difference between the TRL-thickness reduction over the electrodes and over the substrate of groups 2 and 3 at 12 weeks.

### Immunohistochemical analysis

Immunohistochemical examination could not be conducted in group 1 because of severe fibrous reactions and retinal damage at 12 weeks after surgery. 4′,6-diamidino-2-phenylindole (DAPI) staining was used to observe gross cell morphology and implant location. Cells stained with DAPI showed no significant changes compared to the control eye. One specimen in each group was selected to count the number of survived RGCs. There was equivalence between the remaining RGCs of the control, groups 2 and 3, resulting in 50, 46, and 44 cells, respectively.

**FIGURE 5:**
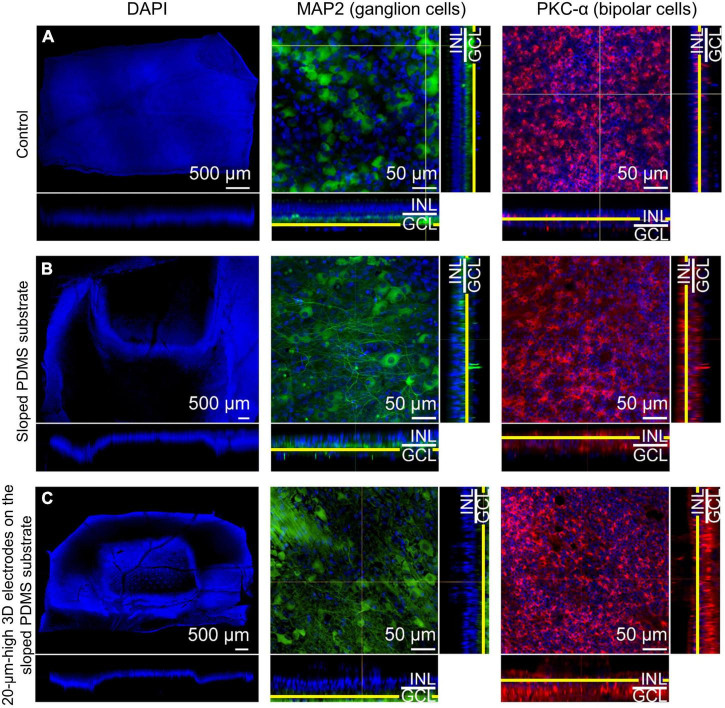
Confocal images of immunohistochemical staining using the tissue-clearing method 12 weeks after surgery. 4′,6-diamidino-2-phenylindole (DAPI) staining (scale bar = 500 μm) was used to observe gross cell morphology and the implant’s location, while microtubule associated protein 2 (MAP2) and protein kinase C-α (PKC-α) were used to observe the ganglion cell layer (GCL) and the bipolar cell layer, respectively for **(A)** the control eye, **(B)** an eye implanted with sloped polydimethylsiloxane (PDMS) substrate (group 2), and **(C)** an eye with 20-μm-high electrodes on a sloped PDMS substrate (group 3). In groups 2 and 3, MAP2 staining in the en-face view indicated that ganglion cells were preserved. Moreover, the three-dimensional (3D) electrodes did not contact the GCL directly on vertical and horizontal dissection. PKC-α staining indicated that the bipolar cell layer was also preserved in both groups.

In group 2, microtubule associated protein 2 (MAP2) staining showed that the RGCs appeared to be well preserved in the en-face view when compared with those in the control eye. Protein kinase C-α (PKC-α) was substantially expressed in the bipolar cell layer, which remained intact. The results also showed that the bipolar cell layer over the electrodes was relatively well maintained in the en-face view, and the electrodes were found not to be in contact with the GCL directly on vertical or horizontal dissection (Figure 5).

**FIGURE 6:**
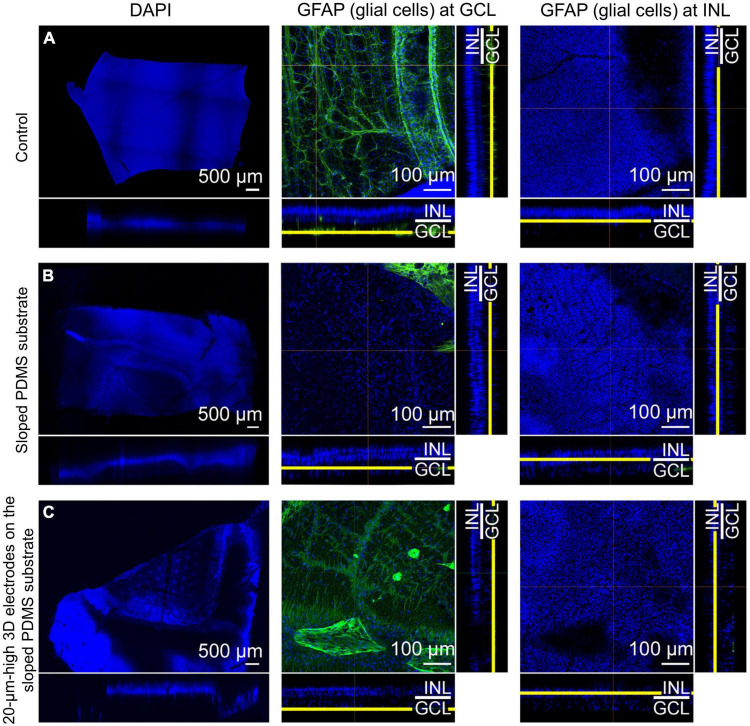
Confocal images of glial fibrillary acidic protein (GFAP) staining using the tissue-clearing method 12 weeks after surgery. Compared to that in the control eye **(A)**, retinal fibrosis in the ganglion cell layer (GCL) was not prominent in groups 2 [sloped polydimethylsiloxane (PDMS) substrate] **(B)** and 3 [20-μm-high three-dimensional (3D) electrodes on the sloped PDMS substrate] **(C)**. Glial proliferation in the inner nuclear layer (INL) was not detected in any group.

In group 3, MAP2 staining indicated that the RGCs appeared to be well preserved in the en-face view when compared with those in the control eye. Similar to group 2, the expression level of PKC-α in the bipolar cell layer was significant and the electrodes did not touch or directly penetrate the GCL on any dissection. Glial fibrillary acidic protein (GFAP) staining did not reveal any prominent retinal fibrosis in the GCL in both groups; moreover, glial proliferation was not detected in the INL in comparison with the control eye (Figure 6). Gliosis may occur at the interface between the implant and the retina. However, as the retina was a thin layer (less than 250 μm thick), if gliosis had occurred, all retinal layers would have been ordinarily affected. Therefore, we concluded no gliosis seemed to appear at the interface between the implant and the retina. In addition, many previous studies have shown that the INL only occasionally displayed weak positive immune fluorescence staining (Jasenka et al., 2009). This might be one of the reasons why it was difficult to observe the INL from these images. For confocal images, GFAP staining was not highlighted with a strong signal. However, this staining could be seen in the peripheral area of the implant, implying that fibrosis did not happen to surround the implant. In addition, GFAP’s expression in pig models might be similar to human species because of the similar cellular anatomy. In humans, GFAP immunolabeling in young and normal specimens was found predominantly in astrocytes in the NFL and GCL (Wu et al., 2003). This may explain why GFAP can be seen predominantly in the second column (the GCL layer) but absent in the third column (the INL layer).

## Discussion

The biocompatibility of subretinal implants is affected not only by the implant material, but also by the implant design and surgical trauma. The present study investigated structural retinal changes after subretinal implantation of electrodes of different designs. Although commercially available subretinal implants such as the Alpha IMS (Stingl et al., 2013b) and Alpha AMS (Edwards et al., 2018) have a thickness of 70 μm and the thickness of the PRIMA photovoltaic subretinal implant is only 30 μm (Lemoine et al., 2020), the maximal tolerable thickness and lateral slope of subretinal implants have not been established. Nevertheless, previous studies have demonstrated that 3D electrodes are generally well-tolerated and that retinal cells in the INL migrate into the space between the 3D electrodes with minimal gliosis locally (Butterwick et al., 2009; Djilas et al., 2011; Bendali et al., 2015; Flores et al., 2018, 2019; Losada et al., 2019; Chen et al., 2020).

Pig eyes are a good model for human eyes when considering implant designs because they are similar in terms of size, retinal physiology, and pathophysiology (Hein et al., 2012; Lim et al., 2018). As the pig retina receives both choroidal and retinal arterial blood, the inner retina remains stable after subretinal implantation. In one of the three mini pigs in group 2, retinal detachment was observed on one side of the implant 2 weeks after surgery; however, this phenomenon resolved spontaneously within 1 month. Neurosensory retinal detachment with other subretinal implants was also reported in a previous study by Adekunle et al. (2015). They reported that such surgically induced retinal detachments resolved within 1 week, and that the retinal vasculature overlying the implant appeared normal on fluorescein angiography. Muqit et al. (2020) also reported adverse effects of retinal detachment 6 weeks after surgery in one eye in a study evaluating surgical techniques for subretinal implantation of PRIMA microchips of two sizes in two different (feline and non-human primate) animal models. They indicated that retinal detachment is a recognized risk in vitreoretinal surgery, and that retinal detachment in similar subretinal surgery models occurs at a rate of 6–9% (Guthoff and Schrader, 2004; Muqit et al., 2020).

Electrode arrays typically consist of a sandwich of several layers of polymer and metal. The polymer can be polyimide, parylene, or silicone (Weiland et al., 2009). Polyimide is widely used for thin electrode arrays in retinal implants (Kim et al., 2009). However, for softness and flexibility, parylene or PDMS are good alternatives that also show excellent long-term results in terms of biocompatibility (Weiland et al., 2009). Because of the retinal curvature, subretinal implants should be within a limited range of sizes and thicknesses to avoid creating a large space between the electrodes and the retina. Previous studies have reported that implants need to have reduced in thickness to achieve higher biocompatibility with retinal tissue (Adekunle et al., 2015; Flores et al., 2018). In addition, subretinal implants need to be placed as close to nerve cells as possible to achieve a lower activation threshold and more selective stimulation of a small group of cells, which can lead to better image resolution. Stimulation is usually transferred to the retina by electrodes, and several previous studies have evaluated the integration of retinal tissue with 3D implants with varying electrode shapes and heights (Butterwick et al., 2009; Flores et al., 2018; Losada et al., 2019).

In the current study, notable post-operative changes in retinal tissue stability were observed in group 1. The implant in this group consisted of a thick substrate with a steep 90° angle at the edge with higher electrodes. According to our normative data for each retinal layer, the total retinal thickness in mini pigs was approximately 180–240 μm; the total mean thickness of the IPL, INL, and ONL was about 75–100 μm; and the mean thicknesses of the outer plexiform layer (OPL) and INL were approximately 18–24 μm and 18.5–24 μm, respectively, depending on different locations (Choi et al., 2021). The total retinal thickness was reported to be approximately 210 μm in wild-type mice and 100 μm in rd10 mice by Pennesi et al. (2012), who also suggested that the region of interest for penetrating microelectrode arrays inside the retina extended from the NFL to the outer margin of the INL (∼100 μm) (Pennesi et al., 2012; Lim et al., 2018). Hence, in this group, we designed the implant with a total height in the areas without electrodes of approximately 240 μm. Our purpose was to maximize the substrate thickness within these normal retinal thickness limits, and we synthesized 75-μm-high electrodes with the aim of stimulating RGCs directly from the subretinal space. Our results showed that from the second week onward, the steep 90° angle at the edge of the implant base appeared to exert physical pressure on the overlying retina, resulting in whole retinal thinning over the implant base, which was more obvious at the substrate edge. The TRL thickness over the substrate and at the substrate edge decreased constantly over the 12-week post-surgery period. In some cases, the retinal detachment and severe fibrosis occurred after surgery (especially in group 1), and thus, the TRL thickness could not be determined over time. The resultant findings, therefore, would need to be considered as limited to only a small number of implanted devices over 12 weeks following surgery. Furthermore, the 3D electrodes with a 75-μm height progressively penetrated the INL and reached the NFL, leading to inner retinal atrophy. Such degeneration would be disadvantageous for functional implant signaling in the remaining retina in the long term. Flores et al. (2018) previously reported that in rats with no visible gliosis, 10-μm-high pillar electrodes reached the middle of the INL, while 22-μm-high pillar electrodes reached the upper portion of the INL; moreover, retinal tissue migrated into the space between the pillar electrodes. Although Chen et al. (2020) reported that 128 μm high electrodes on a 13-μm-thick polyimide substrate could be positioned at the junction of the IPL and GCL without significant gliosis in mini pigs, our experimental group 1 showed that 75-μm-high 3D electrodes reached the top of the GCL. The difference between our results and those of Chen et al. (2020) might have been due to the disparity in substrate thickness (200 vs. 13 μm). The significantly thicker and steeper substrate in our experiment caused diffuse whole-retinal-layer atrophy; thus, even a height of 75 μm was sufficient to penetrate the whole retina.

Although the subretinal implants with a thick PDMS substrate in group 1 showed poor outcomes and resulted in retinal damage, such as INL penetration and severe fibrosis, PDMS can still be used as a base for the electrode arrangement. Moreover, in terms of surgical handling, the implant was reported to be the most compliant and caused the least damage to the retina in a case of incidental contact with the array (Weiland et al., 2009). Therefore, in groups 2 and 3, we modified the PDMS substrate as well as some other factors to mitigate the disadvantages of group 1 implant. Based on the results of group 1 as a preliminary experiment, we adjusted the substrate thickness from 246 to 186 μm, and the slope of the implant sides was changed from 90 to 25–40°. Because we wanted to investigate the stress-relieving effects of the sloped-implant-side design on the overlying retina, we kept the subretinal implant thickness relatively high with respect to the lower limit of the total normal retinal thickness (∼180 μm). The electrode height was decreased to 20 μm. We expected the central portion or the highest point of the electrodes to be in contact with the layer of the INL, and the peripheral portion of the electrodes to be completely covered by the remaining INL. We did not consider OPL, ONL, or PRL thickness when deciding the electrode height because these layers over subretinal implants normally disappear after subretinal prosthesis implantation.

In group 2, the implants consisted of a 186-μm-thick sloped substrate without any 3D electrodes, while the implants in group 3 consisted of 20-μm-high 3D electrodes on a 186-μm-thick sloped base. As expected, the structural compatibility with the retinal tissue was better in groups 2 and 3 than in group 1. SD–OCT images showed that the 20-μm-high 3D electrodes penetrated the retina only until they reached the lower portion of the INL and did not penetrate further. Although TRL thickness over the substrate and at the substrate edge in groups 2 and 3 continuously decreased after 2 weeks post-surgery during the 3-month observation period, this decrease appeared to be much less severe than in group 1. TRL-thickness-loss was observed even in group 2, which received only the PDMS substrate without electrodes. Based on the similar trends of TRL-thickness changes in groups 2 and 3, electrode height was considered not to affect the surrounding retinal layer status significantly as long as it was within certain limits. For a subretinal implant targeting bipolar cells in the INL, the amount of tissue (maximal cell density) remaining above the electrode surface is important. According to a previous study (Lorach et al., 2015a), as electrode height increased, INL thickness over the electrodes decreased. However, it should be noted that insufficient 3D electrode height may result in a loss of advantageous properties such as increased surface area and reduced charge density. Therefore, it is necessary to determine the appropriate height of electrodes considering the remaining thickness of the INL. Xie et al. (2018) reported that the average INL thickness in the porcine retina was approximately 40 μm while our previous study showed that the mean thickness of the INL was approximately 18–24 μm (Choi et al., 2021). Additionally, because retinal cells in the INL are the primary target of subretinal stimulation, an electrode height of less than 40 μm may be reasonable for improving tissue apposition between INL cells and subretinal electrodes with minimal tissue disruption in the degenerated retina.

Moreover, in groups 2 and 3, the substrate was thinner and the edge of the implants was sloped, which resulted in substantially less damage to the inner retinal tissue than in group 1, even though the outer retina, including the PRL and ONL, disappeared. It is possible that the subretinal implants blocked the metabolic interactions between the retina and RPE cells, leading to outer retinal cell apoptosis. Although the substrates of groups 2 and 3 were not as thin as Alpha AMS or PRIMA (Stingl et al., 2017; Palanker et al., 2020), implants in both groups 2 and 3 were stably maintained in the subretinal space over the 12-week experimental period. In addition, the decreases in TRL thickness over the implant and at the substrate edge in both groups were not as severe as in group 1. Especially, it was noteworthy that the steep edge of the implant was more influential than the implant thickness in retinal thinning. In the case with a sloped PDMS substrate implanted upside down under the retina, the retinal tissue was affected differently at both edges of the inverted substrate: the left lateral edge did not damage the retinal tissue, while the right lateral edge penetrated the retinal tissue because of the steep angle along the retinal curvature. Therefore, we suggest that adding a slope to the lateral edge of the implant could be an important approach to enhance biocompatibility by compensating for the stress on the retinal tissue due to the implant height. A sloped substrate edge is essential for preserving the overlying retinal tissue if the thickness of the implant needs to be relatively large.

The present study had a few limitations. First, parylene-C was coated on the entire implants to investigate how different structures of subretinal implants would affect retinal cells. By coating the entire implant using a single biocompatible material, parylene-C, we could eliminate the material factors that could cause possible changes of retinal cells such as fibrosis, leaving only structural factors to be investigated. Nevertheless, to deliver electrical stimulation to retinal cells, electrode tips must be exposed without parylene-C. In our previous studies (Seo et al., 2019, 2020), 3D electrodes were exposed, and the used electrode metal was Pt. The same metal was used on the electrode tips (groups 1 and 3) in the present study. However, there are better metals than Pt for stimulation. For instance, low impedance and high charge injection capacity (CIC) of iridium oxide (IrOx) make it a promising stimulation material (Negi et al., 2010). IrOx shows stable biocompatibility and stimulation performance in *in vivo* environments (Flores et al., 2018; Ho et al., 2019). We are currently developing improved electrodes by applying IrOx for better stimulation performance. Thus, the implant with exposed IrOx electrodes may show good stimulation performance with stable biocompatibility. In addition, the change in TRL thickness over electrode was the least in group 3 over time (Figure 4). Assuming that IrOx electrodes were used in group 3, the results (the TRL thickness over electrode) would not change even if the electrode tips were exposed. Second, as retinal changes were investigated using implants with different parameters, such as electrode height and substrate thickness, accurate comparisons of each design parameter was difficult. However, despite such difficulties in controlling design parameters, we were able to observe the effects of substrate thickness, the angle of the substrate edge, and electrode height. Third, the residual retinal cell density over the implants was not statistically analyzed, because it was not possible to separate the tissue from the implant or to vertically dissect them together at the electrode without damaging the retina for the purposes of conventional histologic examination. Nonetheless, by adopting the tissue-clearing method, which is a useful technique for examining alterations in retinal tissue and their relationships with the subretinal implant in any direction, including the en-face view, we were able to assess the biocompatibility of different electrode designs. Lastly, our study was performed on healthy pigs without retinal degeneration, which would be another limitation. The biocompatibility of subretinal implants has been investigated in previous studies using animal models other than mini pigs. Peyman et al. (1998) reported fibrosis and significant loss of retinal cells, especially the outer retina in areas over the implant in rabbits. This may be predictable because rabbits do not have retinal circulation. In pig models used in the present study, even if the implants were in contact with the degenerated retina, it does not mean that the INL and bipolar cells would degenerate because pigs have both choroidal and retinal arterial blood supply to the retina, unlike rabbits. In our previous study (Choi et al., 2021), the mean thicknesses of the IPL and INL were approximately 46.03 ± 8.95 μm and 20.73 ± 6.59 μm, respectively. Thus, even considering the implants in contact with the degenerated retina, it is expected that 20-μm-high electrodes such as in group 3 would not penetrate into the GCL in most pig eyes. More importantly, we could assess and compare the different implants that affected nourishment to pig eyes which have healthy outer and inner retinal layers. In addition, the eye size of pigs similar to that of humans is an advantage for creating the design of implants suitable for human applications.

## Conclusion

In this study, we evaluated the structural changes in the retina due to various subretinal implants differing in implant geometry. While the 75-μm-high 3D electrodes on a right-angled substrate could not be stably maintained in the retina, subretinal implants with 20-μm-high 3D electrodes on a substrate with sloped sides were stably maintained without damaging the inner retina. To develop more versatile and biocompatible subretinal implants, strategies targeting the implant design, such as approaches considering the height of the electrodes and sloped substrate edges, should be adopted.

## Data availability statement

The original contributions presented in the study are included in the article/Supplementary material, further inquiries can be directed to the corresponding authors.

## Ethics statement

The animal study was reviewed and approved by the Institutional Animal Care and Use Committee of Korea University School of Medicine.

## Author contributions

QV: methodology, validation, investigation, and writing − original draft. HS: conceptualization, methodology, investigation, visualization, and writing − original draft. K-EC: methodology, validation, and formal analysis. NK: methodology and investigation. YK: methodology. JL and S-HP: formal analysis. JK: methodology and formal analysis. SK: conceptualization, methodology, resources, writing − original draft, supervision, project administration, and funding acquisition. S-WK: conceptualization, methodology, validation, resources, writing − original draft, supervision, project administration, and funding acquisition. All authors contributed to the article and approved the submitted version.
