# Supplementary material for: Structural changes in the retina after implantation of subretinal three-dimensional implants in mini pigs
Source: Front Neurosci. 2022 Sep 30;16:1010445. doi: 10.3389/fnins.2022.1010445 (PMC9561346; doi:10.3389/fnins.2022.1010445)
Supplement: Supplementary file 1 [file Data_Sheet_1.docx]

Supplementary Material


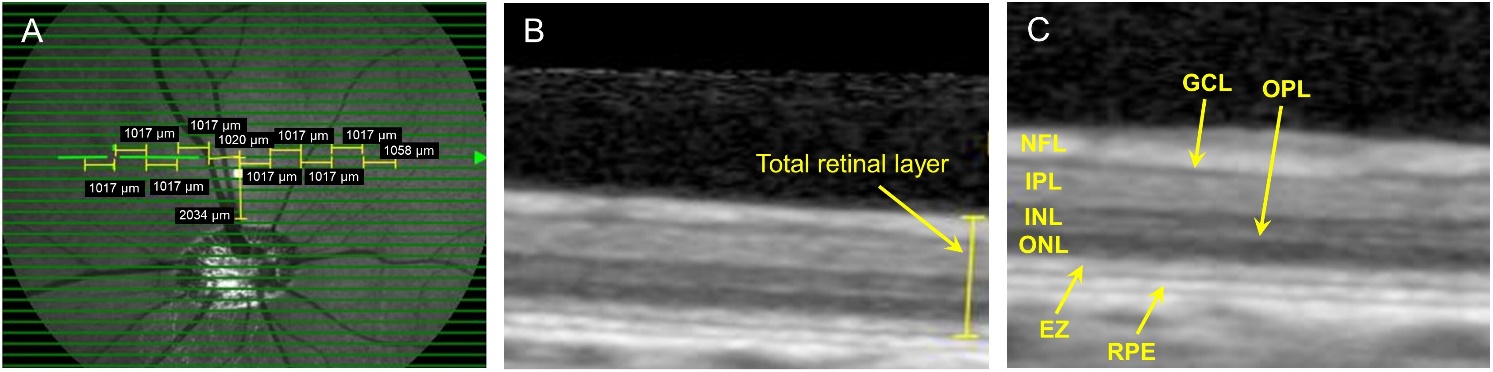


**Supplementary Figure 1.** Total retinal layer (TRL) thickness was measured along a horizontal line perpendicular to the retinal layers in cross-sectional images. TRL was defined as the distance between the inner margin of the internal limiting membrane and the inner margin of the retinal pigment epithelium. **(A)** Before surgery, TRL thickness was measured at 10 points at 1-mm intervals along the visual streak. **(B)** Magnified spectral domain–optical coherence tomography (SD-OCT) image, with the yellow arrow indicating the TRL thickness. **(C)** Magnified SD-OCT image with yellow arrows indicating the individual layers of the retina (NFL: nerve fiber layer; GCL: ganglion cell layer; IPL: inner plexiform layer; INL: inner nuclear layer; OPL: outer plexiform layer; ONL: outer nuclear layer; EZ: ellipsoid zone; RPE: retinal pigment epithelium).


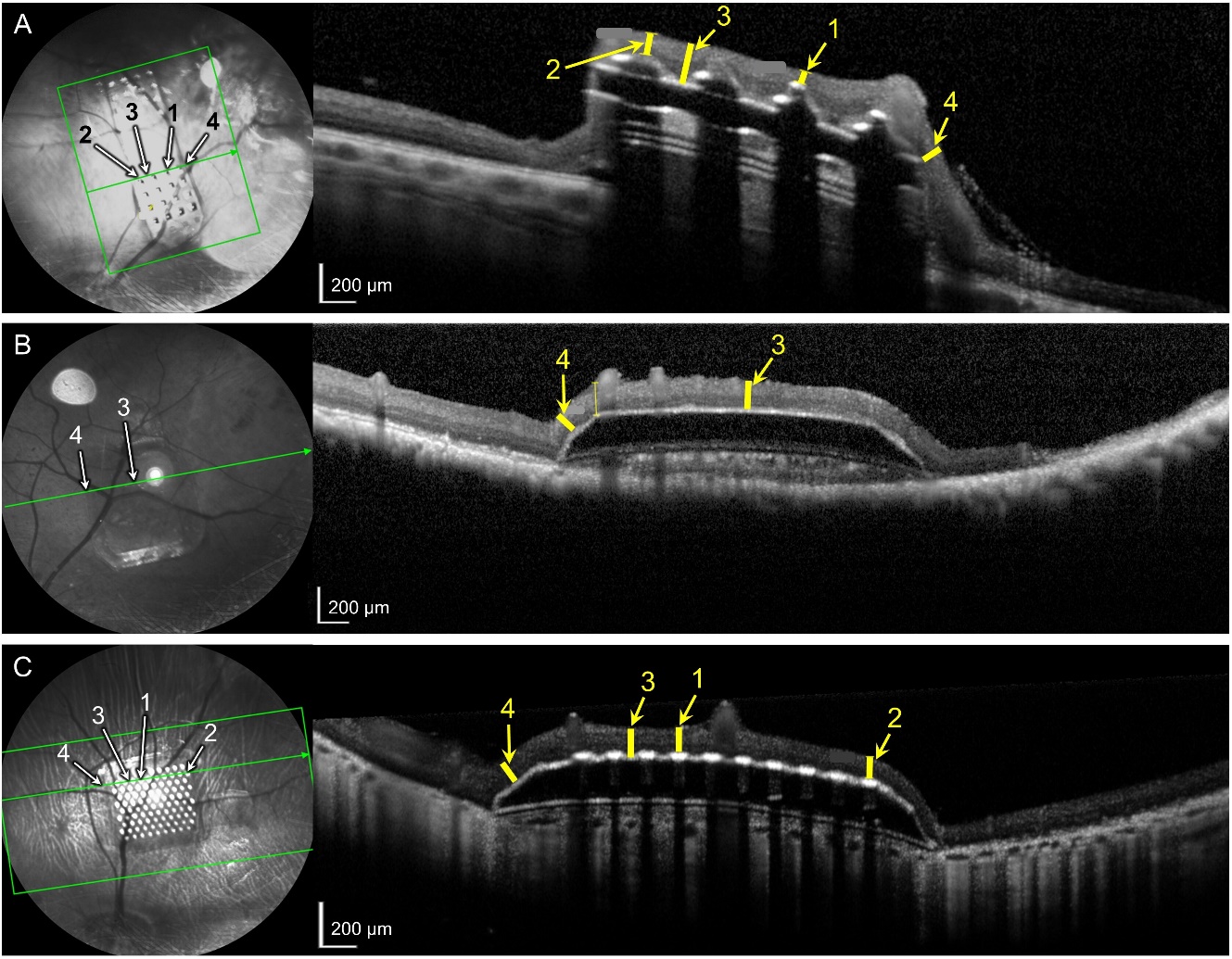


**Supplementary Figure 2.** Total retinal layer (TRL) thicknesses over the electrode (1: center location, 2: marginal location), over the substrate (3), and over the substrate edge (or slope) (4) were measured in **(A)** group 1, **(B)** group 2, and **(C)** group 3. The TRL thickness over the electrode (1, 2) was defined as the perpendicular distance between the center of each electrode and the inner margin of the internal limiting membrane. The TRL thickness over the substrate (3) was defined as the perpendicular distance between the substrate surface (the center points of 2 adjacent electrodes) and the inner margin of the internal limiting membrane. The TRL thickness at the edge or slope of the implant (4) was defined as the perpendicular distance between the edge or sloped surface of the substrate and the inner margin of the internal limiting membrane.


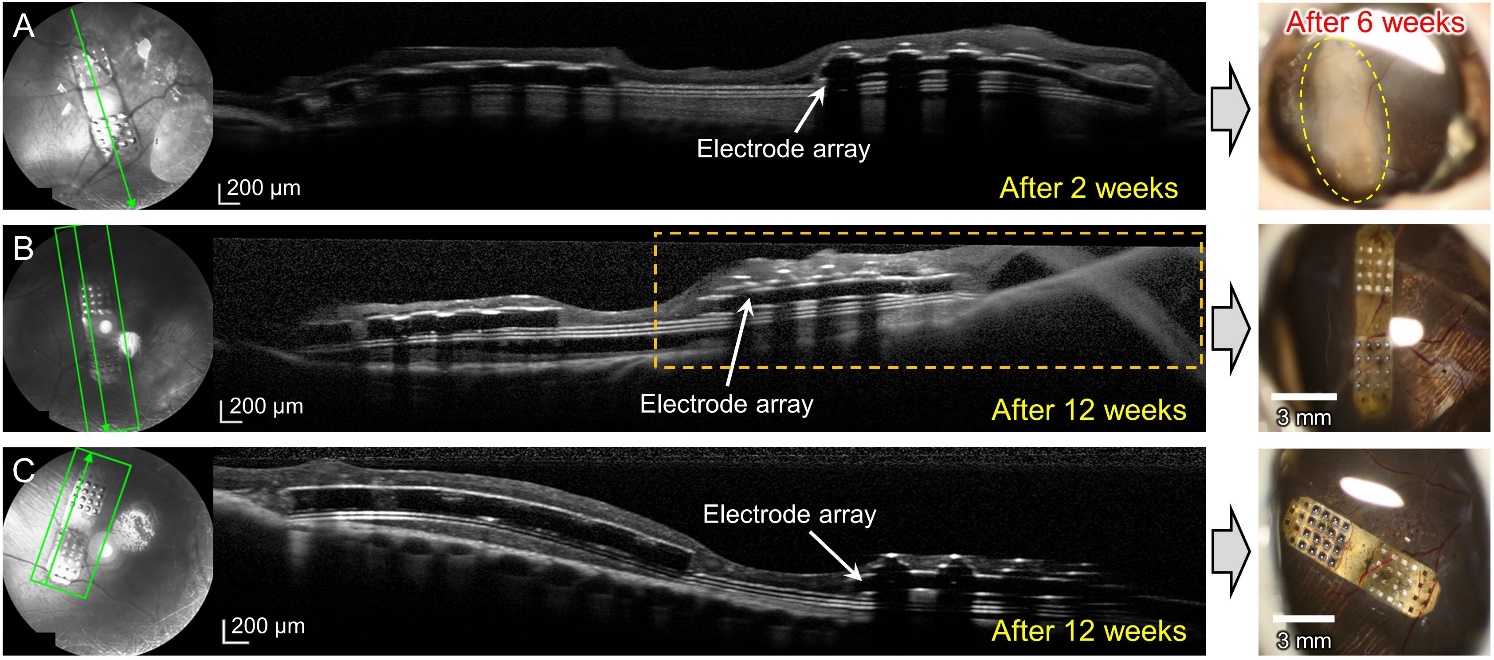


**Supplementary Figure 3.** Spectral domain–optical coherence tomography (SD-OCT) and optical images of group 1 implants with 75-μm-tall 3D electrodes (right) implanted together with a 2D control structure that had the same substrate thickness and right-angled edges but no protruded electrodes (left). The images showed relatively stable integration of the implants with retinal tissue 2 weeks post-implantation in all three pigs. **(A)** In pig 1, the retina was attached and intact without observable fibrosis on SD-OCT images at 2 weeks. However, a severe fibrous reaction was noted around the implant at 6 weeks after enucleation and coronal dissection under the surgical microscope. **(B)** In pig 2, retinal detachment was not detected at 6 weeks but could be observed at 12 weeks. Under the surgical microscope, no severe retinal fibrosis was observed, but the retina was shallowly and locally detached. **(C)** In pig 3, the implant remained stable and undamaged under the retina for 12 weeks. The retina was also found to be attached and intact without fibrosis after enucleation and coronal dissection under a surgical microscope.


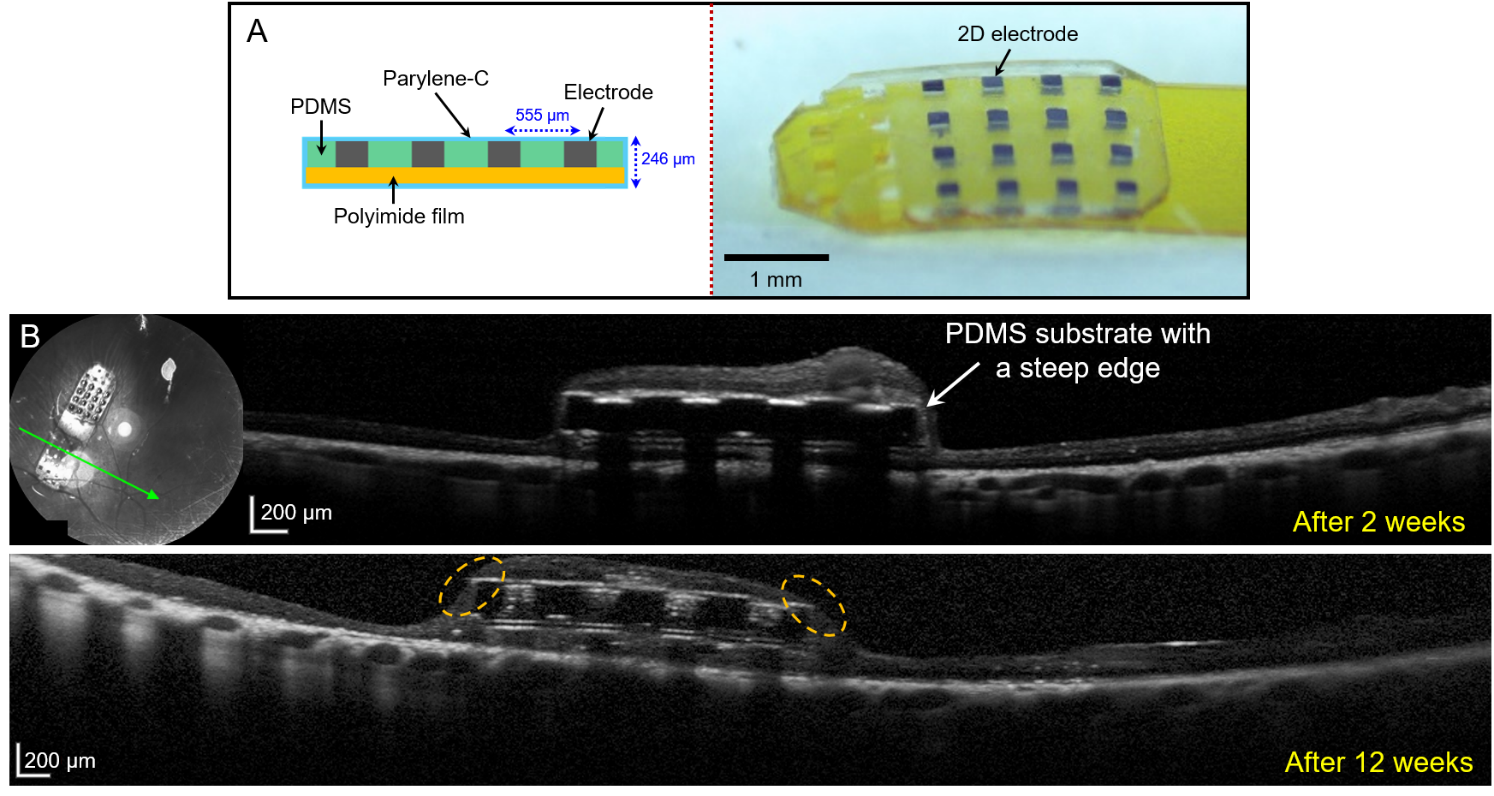


**Supplementary Figure 4. (A)** Two-dimensional (2D) electrodes on a right-angled polydimethylsiloxane (PDMS) substrate. **(B)** Because the steep angle of 90° at the lateral side of the implant (marked by orange dashed circles) exerted a physical force on the retina, retinal thinning was observed not only at the upper edge of the substrate, but also across the substrate, even in the portion without electrodes, from 2 weeks onward.


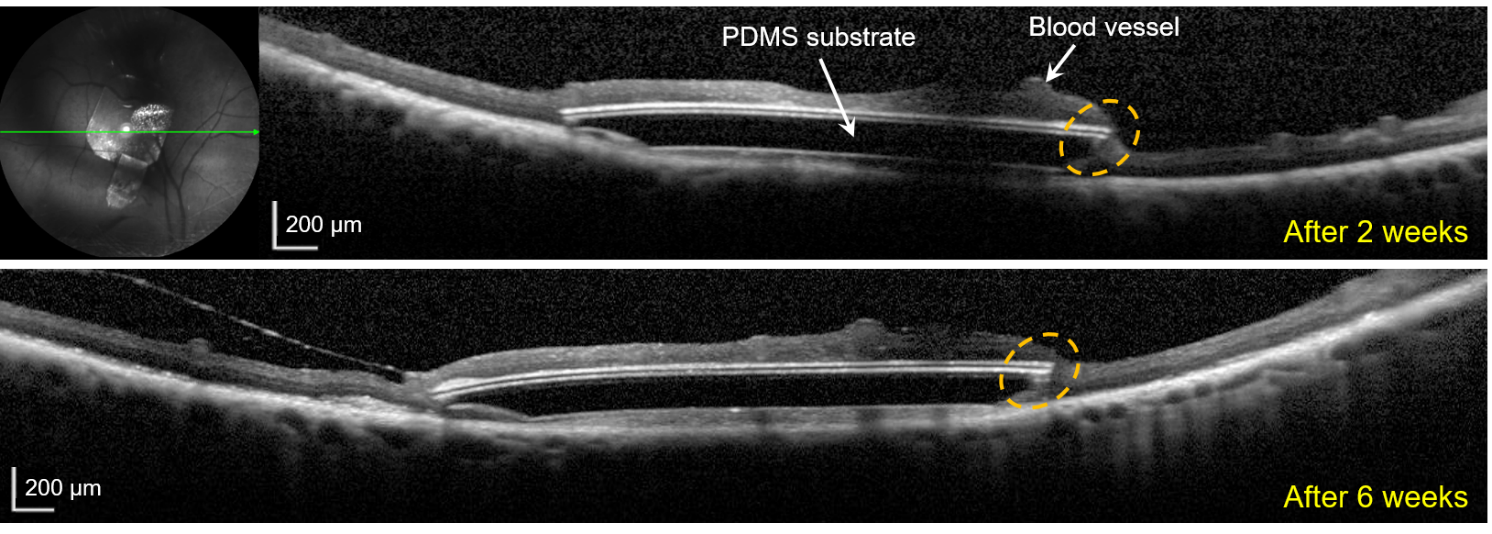


**Supplementary Figure 5.** One of the group 2 implants was implanted upside down due to the surgeon’s error; however, the inverted implant also integrated stably with the retina. The lateral edge of the inverted PDMS substrate on the left side fit well with the curvature of the retina, so the overlying retinal tissue (GCL, inner retina) was not damaged. On the other hand, the lateral edge on the right side nearly penetrated the retinal tissue.
